# A New Benzothiadiazole Derivative with Systemic Acquired Resistance Activity in the Protection of Zucchini (*Cucurbita pepo convar. giromontiina*) against Viral and Fungal Pathogens

**DOI:** 10.3390/plants12010043

**Published:** 2022-12-22

**Authors:** Maciej Spychalski, Rafal Kukawka, Raghavendra Prasad, Natasza Borodynko-Filas, Sylwia Stępniewska-Jarosz, Krzysztof Turczański, Marcin Smiglak

**Affiliations:** 1 Poznan Science and Technology Park, Rubież 46, 61-612 Poznan, Poland; 2 Innosil Sp. z o.o., Rubież 46, 61-612 Poznan, Poland; 3 Environmental Horticulture, Royal Horticultural Society (RHS), Wisley, Surrey GU23 6QB, UK; 4 Plant Disease Clinic and Bank of Pathogens, Institute of Plant Protection-National Research Institute, ul. Węgorka 20, 60-318 Poznan, Poland; 5 Department of Botany and Forest Habitats, Faculty of Forestry and Wood Technology, Poznan University of Life Sciences, Wojska Polskiego 71d, 60-625 Poznan, Poland

**Keywords:** viral diseases, CABYV, WMV, ZYMV, powdery mildew, systemic acquired resistance (SAR), benzothiadiazole, BTHWA

## Abstract

The ability of plant resistance inducers to provide protection against viral diseases is one of their main advantages over conventional pesticides. In the case of viral diseases that cannot be controlled directly with pesticides, insecticides are used to control the vectors of viruses. However, the effectiveness of such treatments is strictly dependent on the time of application. The plant response to the application of systemic acquired resistance (SAR) inducers, as a result of the stimulating action of these substances, does not depend on the time of application as it triggers the plant’s natural defence mechanism. The best-recognised substance showing SAR inducer activity is acibenzolar-S-methyl ester (ASM, BTH). As its activity against different plant pathogens of crops has been well documented, the current research is concentrated on the search for novel substances of the type. The tested substance, *N*-methoxy-*N*-methylbenzo(1,2,3)thiadiazole-7-carboxamide (BTHWA), is an amide derivative of benzothiadiazole, showing plant resistance-inducing activity. This article presents the activity of BTHWA that has led to increased resistance of zucchini (*Cucurbita pepo convar. giromontiina*) towards viral infections. In addition, since the occurrence of the fungal pathogen, powdery mildew, was also observed during the two-year field experiments, the activity of BTHWA related to the reduction of infection with this fungus was also investigated. The substance was applied in two different variants either four or eight times, over the whole vegetation season. Surprisingly, the variant of four applications performed at the beginning of the vegetation season proved more effective in protection against viruses and fungus. A possible explanation may be the occurrence of the growth–immunity trade-off phenomenon that is known in the literature. Disturbance in plant metabolism resulting from eight applications may lead to lower yields of plants treated with SAR inducers. Perhaps such overstimulation of the plants we treated eight times may not have brought the optimum increase in plant resistance.

## 1. Introduction

*Cucurbita pepo convar. giromontiina* is a biologically and economically important crop [1], commonly known as ‘zucchini’ or ‘summer squash’, that belongs to the *Cucurbitaceae* family. It contains a number of beneficial micronutrients such as minerals, carotenoids, vitamin C, and phenolic compounds [2,3]. Zucchini has been used in traditional folk medicine to treat colds and alleviate aches due to its antioxidant, anti-carcinogenic, anti-inflammatory, antiviral, antimicrobial and analgesic activities [3,4,5]. During cultivation, zucchini is exposed to various biotic factors, where plant pathogens cause severe diseases and yield losses.

Viruses are infectious agents that can replicate only inside a living host cell and require vectors to spread or can be easily transmitted with the plant sap. Potyviruses are efficiently transmitted in a nonpersistent manner by many different aphid species. Acquisition and transmission may occur during very short probes (less than a minute), and aphids retain infectivity for a few hours [6]. This group of positive-strand RNA viruses includes, among others, watermelon mosaic virus (WMV), zucchini yellow mosaic virus (ZYMV) and cucurbit aphid-borne yellows virus (CABYV) [7]. WMV induces a diversity of symptoms according to the isolate and the host cultivar. The infected fruits are discoloured and slightly deformed, whereas the infected leaves show mosaic changes, vein banding, leaf deformations, including blisters, and importantly leaf size reduction [8]. The leaves of zucchini attacked with ZYMV show particularly pronounced symptoms including vein clearing, mosaic, yellowing, distortions and severe filiformism. The fruits of infected zucchini plants are generally severely misshaped with prominent knobs [9]. The CABYV-infected leaves are thickened and brittle. The intensity of the above symptoms depends on the type of cultivar and ranges from a yellowing limited to a few older leaves to a complete discolouration of the whole plant [9]. Interestingly, CABYV does not affect fruit quality but leads to flower abortions and a reduction of the number of fruits per plant [10]. Therefore, the fruits collected from infected plants can be readily marketed with no need for a price reduction.

One of the fungal diseases affecting zucchini is powdery mildew caused by *Podosphaera xanthii*. It is one of the most important limiting factors of global cucurbits production [11]. The infection of zucchini with powdery mildew can result in 30–50% yield losses [12]. Disease symptoms are easily recognised by the presence of a visual white powdery mass, mainly composed of mycelia and conidia, on leaf surfaces, petioles, and young stems [13]. When the environmental conditions are favourable for fungus development, fungal colonies may coalesce to cover the entire top surface of the leaves. Premature senesce of infected leaves leads to the loss of foliage and this, in turn, may affect the cucurbit fruits which are not directly or are rarely attacked by powdery mildew fungi. Left without the protection of foliage, they may be malformed, sunburned and ripened prematurely or incompletely [14].

Plant diseases are controlled by agreeable means, such as resistant varieties or plant protection products (PPPs) [15]. In contrast to the pathogens of fungal or bacterial origin, there are no PPPs that can directly combat viral pathogens. Protection against viruses involves the regular use of insecticide treatments aimed at combating their vectors. Another issue to consider when using plant protection products is the risk of acquiring resistance from insects or fungi. To reduce this risk, the active substances of PPPs should be applied in rotation. However, as the number of active substances authorized to trade decreases due to the implementation of Regulation 2015/408/EC8 [16], the possibility of rotational use drops [17]. New chemicals are difficult and expensive to find and develop, moreover, once a new one is in use, pathogens or pests will soon develop resistance to it unless its application is carefully managed. The timeliness of the application of PPPs is an important component of the successful prevention of an epidemic. However, repeated applications of fungicides have often resulted in the development of resistance to the pathogen [18].

The above-mentioned issues are not applicable when using inducers of systemic acquired resistance (SAR), which provide prolonged and broad-spectrum protection on the whole plant level. The induction of SAR involves the generation of signalling molecules at the site of primary infection that is translocated to distal tissues and prepare plants against infection, which involves a cross-talk between various phytohormones, metabolites and proteins [19]. Contrary to conventional PPPs, pathogens do not become resistant to SAR inducers, as their activity is not directed towards them.

Among SAR inducers of chemical origin, benzo(1,2,3)thiadiazole-7-carboxylic acid, S-methyl ester (ASM, BTH), is a well-studied compound [20,21] that has extensively been shown to induce SAR in various crops, such as cucumber [22], strawberry [23], or wheat [24]. The use of the commercially available SAR inducer BTH is limited, mostly because of its poor solubility in water (only 7 mg/L). Much of our earlier efforts have been aimed at obtaining derivatives of benzothiadiazoles with tuned physiochemical and biological properties [17,25,26,27,28,29,30]. The substance being the most effective plant resistance inducer discovered by us is *N*-methoxy-*N*-methylbenzo(1,2,3)thiadiazole-7-carboxamide (BTHWA) which exhibits high inducing efficacy proved at the molecular level [31]. After the treatment, the plants treated with BTHWA solution have shown a higher level of SAR marker genes PAL, NPR1, and PR-1b at 4 h than the untreated control. The above-mentioned marker genes are associated with different signalling pathways (salicylic acid, jasmonate, and ethylene pathway), all in all with a pathogen defence response. It has been observed that the plants treated with BTHWA solution and 7 days after that infected with TMV virus exhibited a lower level of viral RNA accumulation than the controls, which indicated that the viral replication was less efficient. Similar observations were made in the study performed on tobacco leaves infected by the virus prior to the treatment with BTHWA: the viral replication was lowered, which meant that BTHWA had a positive effect on the host plant.

The general aim of this study was to check the use of a novel substance representing benzothiadiazoles, a BTH derivative, namely: *N*-methoxy-*N*-methylbenzo(1,2,3)thiadiazole-7-carboxamide (BTHWA) in protecting zucchini (*Cucurbita pepo convar. giromontiina*) against fungal and viral pathogens in field conditions. During the two-year experiment, both fungal and viral diseases were identified in the plants subjected to the experiment. The use of the tested substance was a supplement to the standard protection program consisting of both fungicide treatments against *Botrytis cinerea* and insecticide treatments against aphids. BTHWA was applied in two different variants of either four or eight times over the whole vegetation season. Plants of untreated control (UTC) treatment variant were not treated with BTHWA.

## 2. Results

**Figure 1:**
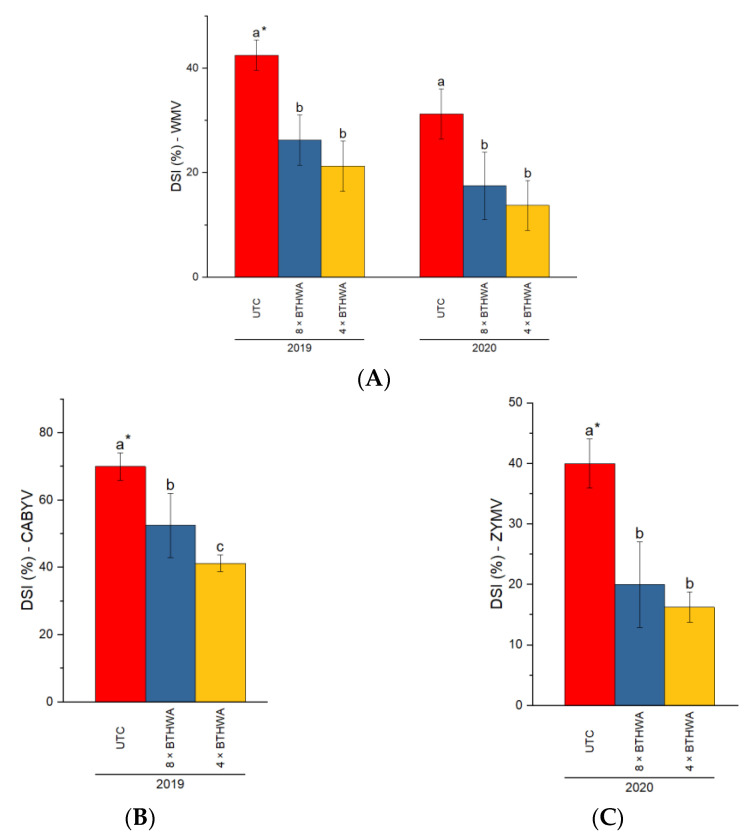
The Disease Severity Index (DSI%) for zucchini (*Cucurbita pepo convar. giromontiina)* infected with WMV (**A**), CABYV (**B**), and ZYMV (**C**). Statistical differences appeared between the plants subjected to different variants of treatments in respective years (treatment × years). * means followed by different letters indicates a statistically significant difference at *p* < 0.05 according to Tukey’s HSD (*n* = 4). UTC—Untreated Control plants that were not treated with BTHWA, 8 × BTHWA—plants that were treated with BTHWA eight times, 4 × BTHWA—plants that were treated with BTHWA four times.

In the study, the Disease Severity Index (DSI) values for three viral pathogens were calculated and presented (Figure 1A–C). 

As for the plants treated with BTHWA, the values of DSI for WMV (Figure 1A) were significantly lower compared to those for the plants of untreated control (UTC) in both years of the study. The lowest DSI was noted for the plants subjected to four treatments, and interestingly it was lower than for the plants subjected to eight treatments with BTHWA, however, this difference was not significant.

Of note, in the experimental years, 2019 and 2020, the viral infection pattern for other identified viruses was quite different. In the first year of the study (2019), ZYMV was not identified in the tested plants, while in the second year (2020) the plants were not observed to be infected with CABYV.

As for the CABYV (Figure 1B), the disease severity of the plants treated with BTHWA was significantly lower, compared to that of the UTC plants. However, the lowest DSI was obtained for the 4 × BTHWA-treated plants, which is statistically significantly lower than that of the 8 × BTHWA-treated plants.

Regarding ZYMV, in 2020, the plants treated with BTHWA in both variants of treatment showed statistically similar results, as represented by close DSI values describing infection with this virus (Figure 1C). However, the 4 × BTHWA treatment can be more effective against ZYMV, as the SD value for the 4 × BTHWA-treated plants was lower than the SD values for the 8 × BTHWA-treated plants, indicating more resistance against this particular viral pathogen.

**Figure 2:**
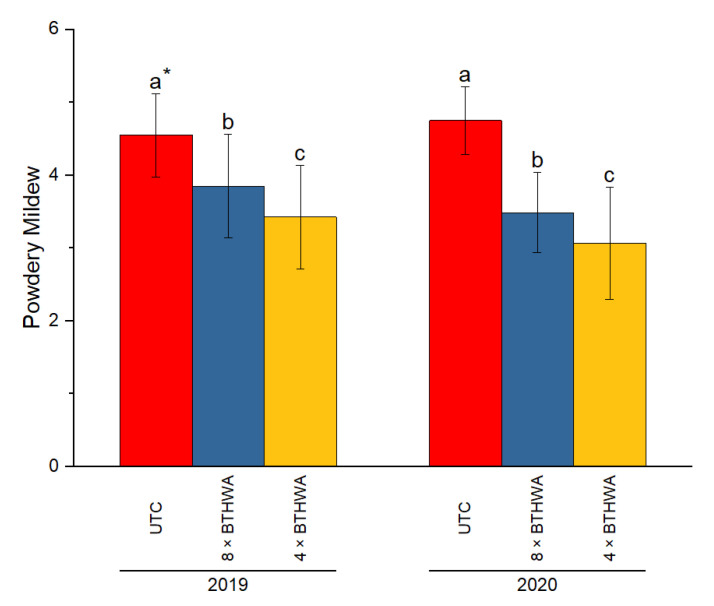
The powdery mildew disease incidence in zucchini (*Cucurbita pepo convar. giromontiina*) was treated in two variants with BTHWA in 2019 and 2020. Significant differences found between the results for the plants treated in the same variant in respective years (treatments × years), * means followed by different letters indicate significant difference at *p* < 0.05 according to Tukey’s HSD (*n* = 20). UTC—Untreated Control plants that were not treated with BTHWA, 8 × BTHWA—plants that were treated with BTHWA eight times, 4 × BTHWA—plants that were treated with BTHWA four times.

**Figure 3:**
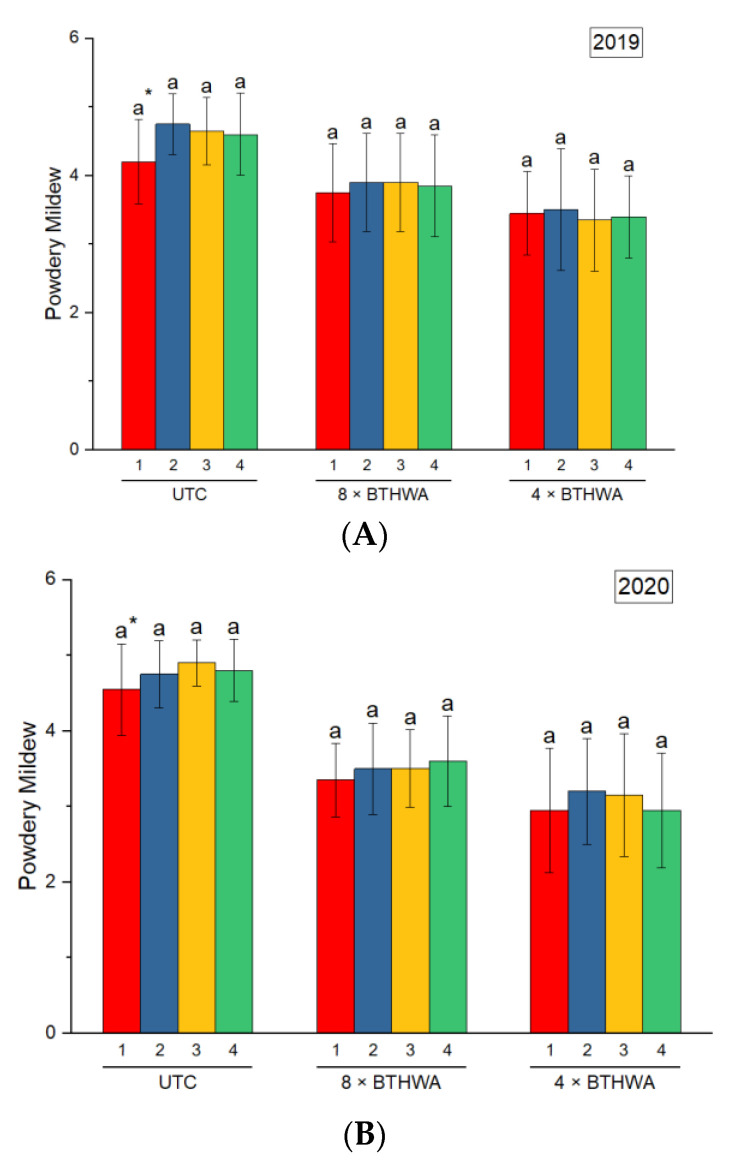
The powdery mildew disease incidence in zucchini (*Cucurbita pepo convar. giromontiina*) for four experimental blocks in response to different variants of treatment with BTHWA in 2019 (**A**) and 2020 (**B**). Statistical differences found between plants in different experimental blocks (treatments × blocks) in respective years, the numbers 1, 2, 3, and 4 represent four blocks. * means followed by different letters indicates significant difference at *p* < 0.05 according to Tukey’s HSD (*n* = 20). UTC—Untreated Control plants that were not treated with BTHWA, 8 × BTHWA—plants that were treated with BTHWA eight times, 4 × BTHWA—plants that were treated with BTHWA four times.

The incidences of powdery mildew in zucchini were recorded for all plants through all studied variants of treatment, and the level of infection was scored from 0–5 according to [32] (Figure 2). The results clearly indicated that BTHWA had a significant influence on the limitation of the level of infection. In both studied years, the level of infection with powdery mildew was the highest in the untreated control (UTC). The variant of treatment consisting of eight applications of BTHWA (8 × BTHWA) resulted in significant limitation of the level of infection, compared to UTC. However, the lowest level of infection was obtained for the variant of treatment with BTHWA applied only four times (4 × BTHWA). The observed results were the same in both studied years. Moreover, the influence of BTHWA in both variants of treatment was consistent for four blocks studied over 2 experimental years, i.e., 2019 (Figure 3A) and 2020 (Figure 3B), which is evidenced by no significant statistical differences between the results for particular variants of treatments.

**Figure 4:**
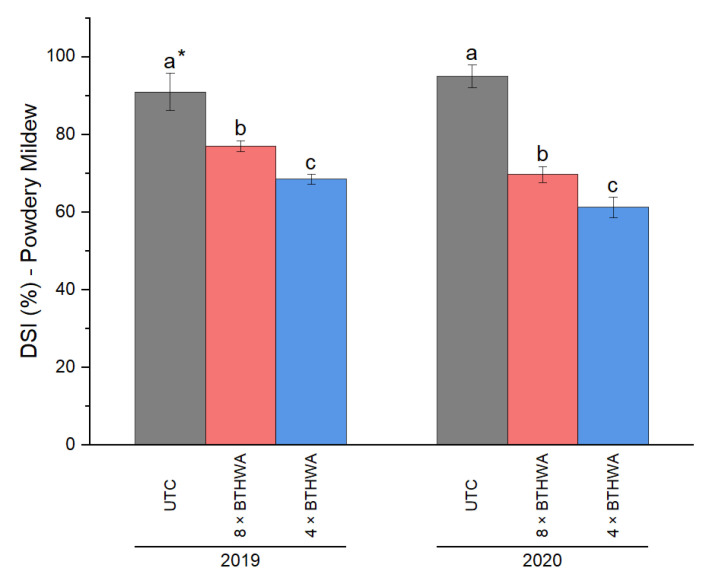
The Disease Severity Index (DSI%) for zucchini (*Cucurbita pepo convar. giromontiina*) infected with powdery mildew. Statistical differences appeared between the plants subjected to different variants of treatments in respective years (treatment × years). * means followed by different letters indicate a statistically significant difference at *p* < 0.05 according to Tukey’s HSD (*n* = 4). UTC—Untreated Control plants that were not treated with BTHWA, 8 × BTHWA—plants that were treated with BTHWA eight times, 4 × BTHWA—plants that were treated with BTHWA four times.

Similarly, as for viral pathogens, the DSI for powdery mildew was also calculated. The DSI for powdery mildew were fully consistent with the previously reported results of scoring powdery mildew incidence. According to the DSI values, the disease severity for the plants of UTC was significantly higher than that of 8 × BTHWA-treated plants and 4 × BTHWA-treated ones (Figure 4). The disease severity was lowered as a result of BTHWA application, and the lowest powdery mildew DSI was noted in the 4 × BTHWA-treated plants.

## 3. Discussion

Viral diseases have always been a serious problem in agriculture as no effective control strategy has been developed as yet, in particular when the infections already occurred. The management strategies have relied on the control of their vectors and the adoption of cultural measures unfavourable to their development such as the use of isolated plots with a regular inspection of sanitation measures along with weeding [33]. Given the above, the research aimed at the development of novel approaches providing sufficient protection against viruses is of high importance.

The presence of the viruses we studied was reported by the Institute of Plant Protection-National Research Institute (IPP-NRI) in Poland [34,35]. The distribution of particular species may vary over time. According to the IPP studies, in 2012, in Poland, CMV was the most prevalent, whereas, in 2014–2015, increased incidences of WMV were observed. ZYMV was detected in 2012, 2014, and 2015, also in mixed infections with other viruses [35]. In 2018, for the first time, cucurbit aphid-borne yellows virus (CABYV), which is one of the most common cucurbit viruses distributed worldwide, was detected in zucchini crops in Poland [34]. 

Overall, based on the above-presented results, it can be inferred that the application of BTHWA activates the plant’s natural defence mechanism as a result of SAR, creating resistance to both fungal and viral pathogens studied. When it comes to the optimum dose, the results clearly indicated that the 4 × BTHWA application is enough to stimulate the zucchini (*Cucurbita pepo convar. giromontiina*) defence mechanism to fight against the infection with powdery mildew, WMV, CABYV, and ZYMV. Prolonged exposure of the treated plants to the SAR inducers (eight treatments) leads to overstimulation of the plants which diminishes the SAR activity build-up after the first four treatments.

The response of plants to the treatment with SAR inducers in field conditions may vary according to different environmental conditions, genotype, crop nutrition and the extent to which the plants’ resistance has already been induced. Understanding the impact of these factors on the level of plant resistance is still unsatisfactory, even though studies in this area have intensified over the last few years [36].

The induction of SAR is associated with the change in plant resource allocation known as growth–immunity trade-off, which can lead to a reduction in yield. Over the years, the growth–immunity trade-off model has been widely described [37,38,39]. Along with the development of knowledge in this field, the model has become more plastic, in which the processes related to plant growth and resistance were strongly intertwined and highly dynamic [40]. In this study, the aspect related to the yielding of zucchini plants was not investigated as the major pressure was on the SAR induction. However, this issue has to be investigated as it is equally important in the context of the use of BTHWA in agriculture. Such a study is also important due to the fact that in our previous work on strawberries, we observed a much wider effect of BTHWA than just inducing plant resistance. As observed in our other studies performed on strawberries, the effect of BTHWA was much broader than just the induction of plant resistance [41]. It stimulates the vegetative development of plants, but at the same time reduces the intensity of flowering and fruit set. These effects should definitely be taken into account when choosing the right application date, depending on the developmental phase of the plants. Presently, it is supposed that the plants that would produce too many flowers and set too many fruits could be treated in the period of flowering as then not only the SAR mechanism would be activated, but also the number of fruits would be curbed. The above-mentioned observations should certainly be taken into account in further research when planning the field experiments, not only testing BTHWA but also other substances expected to show SAR induction ability. 

Plant response to treatment with SAR inducers should be investigated not only in accordance with the number of treatments, their frequency and concentration of the active substance but also according to the developmental phase of the treated plant. It should also be highlighted that the knowledge gained in this area will most likely not be universal for all plant species.

Our assumptions indicate that such an excessive metabolic imbalance may also be reflected in the level of induction of plant resistance. To verify this hypothesis, it would be necessary to perform molecular studies of plant response in conjunction with, e.g., analysis of the viral RNA accumulation in infected plants treated with different concentrations of active substances and at different time intervals and with a different number of treatments, similarly as in our previous studies [31].

However, the results obtained from the hitherto study indicate that excessive use of SAR inducers, although resulted in providing higher protection compared to untreated control, does not give a proportionally greater effect than a lower number of treatments. Other studies have indicated that greater efficiency in protecting the tomato in the field was achieved after biweekly SAR inducer treatments than after the treatments in weekly intervals [42]. Therefore, in conclusion, to fully exploit the potential of resistance inducers, detailed research should be carried out to consider a large number of different variables that may affect the plant response, which, unfortunately, is much more difficult and cost-consuming than in the case of research of new active substances of plant protection products. 

## 4. Materials and Methods

### 4.1. Tested Substance

The substance studied was a benzothiadiazole derivative obtained in our group: *N*-methoxy-*N*-methylbenzo(1,2,3)thiadiazole-7-carboxamide [42,43], provided by the company Innosil Ltd. (Poznan, Poland). BTHWA was obtained with 99.9% purity and used for the preparation of a suspension concentrate (SC) type formulation. In this formulation, BTHWA occurs in the form of solid particles dispersed in the liquid containing a polymeric dispersant and a thickening agent (a polysaccharide), whose presence is to ensure homogeneous dispersion after shaking. The SC-type formulation we used contained 10 g of BTHWA in 1 L and was added to the working solution to get the concentration of 20 mg/L. 

### 4.2. Experimental Design

A field experiment was conducted at the private experimental field in Wielowies in Poland (52°48′ N, 18°07′ E, 81 m above sea level) in two consecutive years, 2019 and 2020, on the cultivation of zucchini (*Cucurbita pepo convar. giromontiina*). The seeds were sown in the second week of April 2019 and 2020. The use of the BTHWA was a supplement to the standard protection program consisting of both fungicide treatments against *Botrytis cinerea* and insecticide treatments against aphids. The substance was applied in two different variants of either 4 or 8 every approximately 14 days, depending on weather conditions. In the first variant, including 4 treatments (4 × BTHWA), treatments were performed in the first 4 dates, while in the second tested variant, consisting of 8 treatments (8 × BTHWA), a total of 8 treatments were performed during the entire vegetation cycle. Plants of untreated control variant of treatment were not treated with BTHWA.

Each variant of treatment consisted of 4 blocks organised in a randomised block design manner. Within each block, 20 consecutive plants were scored for the severity of powdery mildew and sampled for viral infection assessment. The treatments with BTHWA supplemented the standard fungicidal and insecticidal program, presented in Tables S1 and S2. The meteorological data during both experimental periods were obtained from the weather station of Wybranowo and are shown in Tables S3 and S4. 

### 4.3. Assessment of Powdery Mildew Infection

The assessment of the disease incidence and severity was made on all plants based on the percentage of infected leaves as well as the percentage of the leaf surface affected by the disease, in consistence with the EPPO Standard scale [32]. The powdery mildew incidence and severity were assessed once on the 10th day after the last application of BTHWA. The mean values for each assessed block are presented in Table S5.

### 4.4. Assessment of Viral Pathogens Infection

Samples were collected 10 days after the last treatment with BTHWA. All samples were individually tested for CABYV, ZYMV, and WMV using the double-antibody sandwich enzyme-linked immunosorbent assay (DAS-ELISA) [44]. Form each plant, the sample consisting of two mature leaves and fruits was taken. The leaves and fruits were homogenised together in a plastic bag (BioReba AG, Reinach, Switzerland) with an extraction buffer (1:10 *w*/*v*) (LOEWE^®^ Biochemica GmbH, Sauerlach, Germany) using a Homex 6 homogeniser (BioReba AG, Reinach, Switzerland). Sap from healthy plants was used as a negative control. Viruses maintained under greenhouse conditions were used as positive control. The analysis was performed on two replicates per sample.

The DAS-ELISA test was performed following the manufacturer’s instructions, using CaBYV, ZYMV, and WMV, 100 μL of the plant sap and specific diagnostic kits from DSMZ (Braunschweig, Germany) and Loewe Biochemica GmbH (Sauerlach, Germany) according to the manufacturers’ recommendations. The absorbance values (A405) were measured using an immune plate reader (BioTek Instruments, Winooski, VT, USA). The recorded values in the infected samples were more than three times higher than the values of the healthy control plants. The mean values for each assessed block are presented in Table S5.

### 4.5. Assessment of Pathogens Infection

The degree of infection with powdery mildew as well as the percentage of plants infected with investigated viruses was assessed as described above. However, a different approach for expressing such data is the use of the disease severity index (DSI) [45,46]. The DSI is a single index number for summarising the total effect of the disease on a single plant or a small sample of plants. Mean values for each assessed block are presented in Table S5, and subsequent calculation was made according to the equation:

$$\mathrm{DSI}\;\left[\%\right]\frac{\sum \left(class\;frequency\;x\;score\;of\;rating\;class\right)}{\left(total\;number\;of\;observations\right)\;x\;\left(maximal\;disease\;index\right)}\times 100$$

### 4.6. Statistical Analysis

The data were analysed from 4 blocks with 20 replicates each. The presented parameters are means ± Standard Deviation (SD) of 20 plants (*n* = 20), while the calculated Disease Severity Index (DSI) values are given as mean ± SD of 4 blocks (*n* = 4). All recorded and calculated data were evaluated by analysis of variance (ANOVA), and the mean differences were compared by post hoc test at a *p* < 0.05 level, according to Tukey’s HSD. Statistical analyses were performed using OriginLab 2022 software for Windows (OriginLab Corp., Northampton, MA, USA).

## Data Availability

The data presented in this study are available on request from the corresponding author.

## Supplementary Materials

The following supporting information can be downloaded at: https://www.mdpi.com/article/10.3390/plants12010043/s1: Table S1. Schedule of treatments for the experiment in 2019; Table S2. Table S2 Schedule of treatments for the experiment in 2020; Table S3. Table S3 Characteristics of atmospheric conditions during the experiment in 2019; Table S4. Table S4 Characteristics of atmospheric conditions during the experiment in 2020 and Table S5. Table with mean values obtained for powdery mildew assessment and DSI calculation and for DSI values calculated viral pathogens.
