# Supplementary material for: A New Benzothiadiazole Derivative with Systemic Acquired Resistance Activity in the Protection of Zucchini (Cucurbita pepo convar. giromontiina) against Viral and Fungal Pathogens
Source: Plants (Basel). 2022 Dec 22;12(1):43. doi: 10.3390/plants12010043 (PMC9823545; doi:10.3390/plants12010043)
Supplement: Supplementary file 1 [file plants-12-00043-s001.zip › plants-2041181-supplementary.pdf]

Supplementary Materials

A new benzothiadiazole derivative with systemic acquired resistance activity in the protection of Zucchini (*Cucurbita pepo convar. giromontiina*) against viral and fungal pathogens

Maciej Spychalski [1], Rafal Kukawka [1,2], Raghavendra Prasad [3], Natasza Borodynko-Filas [4], Sylwia Stępniewska-Jarosz [4], Krzysztof Turczański [5] and Marcin Smiglak [1,2*]

[1] Poznan Science and Technology Park, Rubież 46, 61-612 Poznań, Poland; maciej.spychalski@ppnt.poznan.pl (M.Sp.); kukawka.rafal@gmail.com (R.K.); marcin.smiglak@ppnt.poznan.pl (M.S.)

[2] Innosil Sp. z o.o., Rubież 46, 61-612 Poznań, Poland; biuro.innosil@gmail.com

[3] Environmental Horticulture, Royal Horticultural Society (RHS), Wisley, Surrey, United Kingdom

[4] Plant Disease Clinic and Bank of Pathogens, Institute of Plant Protection-National Research Institute, ul. Węgorka 20, 60-318 Poznań, Poland, n.borodynko@iorpib.poznan.pl (N.B-F.), s.jarosz@iorpib.poznan.pl (S.S-J.)

[5] Department of Entomology and Forest Phytopathology, Faculty of Forestry and Wood Technology, Poznań University of Life Sciences, Wojska Polskiego 71c, 60-625 Poznań, Poland; krzysztof.turczanski@up.poznan.pl

Table of content

Table S1 Schedule of treatments for the experiment in 2019 3

Table S2 Schedule of treatments for the experiment in 2020 4

Table S3 Characteristics of atmospheric conditions during the experiment in 2019. 5

Table S4 Characteristics of atmospheric conditions during the experiment in 2020. 5

Table S5 Table with mean values obtained for powdery mildew assessment and DSI calculation and for DSI values calculated viral pathogens 6

Table S1 Schedule of treatments for the experiment in 2019

Variants of treatment	Date of treatments							
	10.06.2019	17.06.2019	21.06.2019	24.06.2019	1.07.2019	8.07.2019	10.07.2019	15.07.2019
Untreated Control (UTC)		EMULPAR 940 EC		SWITCH 62,5 WG		SWITCH 62,5 WG	SILTAC EC	BTHWA 10 SC
4 treatments with BTHWA (4xBTHWA)	BTHWA 10 SC	EMULPAR 940 EC	BTHWA 10 SC	SWITCH 62,5 WG	BTHWA 10SC	SWITCH 62,5 WG	SILTAC EC	BTHWA 10 SC
8 treatments with BTHWA (8xBTHWA)	BTHWA 10 SC	EMULPAR 940 EC	BTHWA 10 SC	SWITCH 62,5 WG	BTHWA 10SC	SWITCH 62,5 WG	SILTAC EC	BTHWA 10 SC

Variants of treatment	Date of treatments						
	20.07.2019	29.07.2019	5.08.2019	10.08.2019	15.08.2019	24.08.2019	06.09.2019
Untreated Control (UTC)	EMULPAR 940 EC		BIOSEPT ACTIVE		SILTAC EC		
4 treatments with BTHWA (4xBTHWA)	EMULPAR 940 EC		BIOSEPT ACTIVE		SILTAC EC		
8 treatments with BTHWA (8xBTHWA)	EMULPAR 940 EC	BTHWA 10 SC	BIOSEPT ACTIVE	BTHWA 10SC	SILTAC EC	BTHWA 10SC	BTHWA 10SC

FUNGICIDES: Switch 62,5 WG (cyprodinil 37,5%, fludioxonil 25%; Syngenta Polska, 1kg/ha, 600L/ha) against *Botrytis cinerea*; Biosept Active (extract from grapefruit 25L/ha, 600L/ha) against *Botrytis cinerea*; **INSECTICIDES**: EMULPAR 940 EC (Target, 6l/ha, 500L/ha) against aphids , SILTAC EC (ICB-Pharma, 500ml/ha, 400 l/ha) against aphids; **SYSTEMIC ACQUIRED RESISTANCE INDUCERS**: BTHWA 10SC (*N*-methoxy-*N*-methylbenzo(1.2.3)thiadiazole-7-carboxamide 1%, Innosil Sp. z o.o., 1l/ha, 400l/ha)

Table S2 Schedule of treatments for the experiment in 2020

Variants of treatment	Date of treatments							
	8.06.2020	17.06.2020	20.06.2020	27.06.2020	1.07.2020	7.07.2020	14.07.2020	17.07.2020
Untreated Control (UTC)		EMULPAR 940 EC		SWITCH 62,5 WG		SWITCH 62,5 WG	SILTAC EC	BTHWA 10 SC
4 treatments with BTHWA (4xBTHWA)	BTHWA 10 SC	EMULPAR 940 EC	BTHWA 10 SC	SWITCH 62,5 WG	BTHWA 10SC	SWITCH 62,5 WG	SILTAC EC	BTHWA 10 SC
8 treatments with BTHWA (8xBTHWA)	BTHWA 10 SC	EMULPAR 940 EC	BTHWA 10 SC	SWITCH 62,5 WG	BTHWA 10SC	SWITCH 62,5 WG	SILTAC EC	BTHWA 10 SC

Variants of treatment	Date of treatments						
	22.07.2020	31.07.2020	6.08.2020	9.08.2020	15.08.2020	25.08.2020	7.09.2020
Untreated Control (UTC)	EMULPAR 940 EC		BIOSEPT ACTIVE		SILTAC EC		
4 treatments with BTHWA (4xBTHWA)	EMULPAR 940 EC		BIOSEPT ACTIVE		SILTAC EC		
8 treatments with BTHWA (8xBTHWA)	EMULPAR 940 EC	BTHWA 10 SC	BIOSEPT ACTIVE	BTHWA 10SC	SILTAC EC	BTHWA 10SC	BTHWA 10SC

FUNGICIDES: Switch 62,5 WG (cyprodinil 37,5%, fludioxonil 25%; Syngenta Polska, 1kg/ha, 600L/ha) against *Botrytis cinerea*; Biosept Active (extract from grapefruit 25L/ha, 600L/ha) against *Botrytis cinerea*; **INSECTICIDES**: EMULPAR 940 EC (Target, 6l/ha, 500L/ha) against aphids , SILTAC EC (ICB-Pharma, 500ml/ha, 400 l/ha) against aphids; **SYSTEMIC ACQUIRED RESISTANCE INDUCERS**: BTHWA 10SC (*N*-methoxy-*N*-methylbenzo(1.2.3)thiadiazole-7-carboxamide 1%, Innosil Sp. z o.o., 1l/ha, 400l/ha)

Table S3 Characteristics of atmospheric conditions during the experiment in 2019.

Month/Year	Terce	Mean Temperature [°C]	Sum of Rainfall [mm]	Wind [m/s]
March 2019	1	5.86	10.60	5.16
March 2019	2	5.15	18.30	4.75
March 2019	3	7.45	6.90	3.15
April 2019	1	8.36	0.00	4.45
April 2019	2	7.61	0.10	3.12
April 2019	3	14.01	7.50	4.21
May 2019	1	9.41	4.00	3.08
May 2019	2	12.56	38.70	2.77
May 2019	3	15.41	38.20	2.3
June 2019	1	22.14	1.10	2.4
June 2019	2	22.98	0.80	2.56
June 2019	3	21.3	29.50	2.11
July 2019	1	16.63	0.60	3.05
July 2019	2	18.2	6.20	1.77
July 2019	3	21.8	0.00	2.4
August 2019	1	19.59	18.10	2.13
August 2019	2	19.87	8.10	2.11
August 2019	3	22.22	0.00	1.95
September 2019	1	16.78	26.40	2.52
September 2019	2	12.92	5.40	3.28
September 2019	3	12.63	37.50	2.7

Table S4 Characteristics of atmospheric conditions during the experiment in 2020.

Month/Year	Terce	Mean Temperature [°C]	Sum of Rainfall [mm]	Wind [m/s]
March 2020	1	4.17	15.00	2.79
March 2020	2	6.27	11.40	3.96
March 2020	3	2.98	0.00	3.66
April 2020	1	7.88	0.00	3.37
April 2020	2	7.38	2.20	3.49
April 2020	3	11.24	0.20	2.85
May 2020	1	11.88	8.00	2.92
May 2020	2	10.61	0.10	2.97
May 2020	3	12.37	0.00	2.97
June 2020	1	15.20	0.00	2.75
June 2020	2	20.23	2.20	2.14
June 2020	3	15.24	58.40	2.23
July 2020	1	17.02	15.80	2.39
July 2020	2	18.45	33.30	1.96
July 2020	3	19.51	1.30	1.85
August 2020	1	20.94	0.90	1.57
August 2020	2	21.01	1.30	1.62
August 2020	3	16.93	26.40	2.72
September 2020	1	14.61	31.40	2.19
September 2020	2	15.67	0.00	1.95
September 2020	3	15.06	47.40	2.54

Table S5 Table with mean values obtained for powdery mildew assessment and DSI calculation and for DSI values calculated viral pathogens

Year	Variant of Treatment	Number of block	DSI Index for WMV	DSI Index for CABYV	DSI Index for ZYMV	Powdery mildew assessment	DSI index for Powdery Mildew
2019	UTC	1	0.45	0.65	none	4.2	0.84
2019	UTC	2	0.4	0.75	none	4.75	0.95
2019	UTC	3	0.45	0.7	none	4.65	0.93
2019	UTC	4	0.4	0.7	none	4.6	0.92
2020	UTC	1	0.35	none	0.4	4.55	0.91
2020	UTC	2	0.3	none	0.45	4.75	0.95
2020	UTC	3	0.35	none	0.35	4.9	0.98
2020	UTC	4	0.25	none	0.4	4.8	0.96
2019	8xBTHWA	1	0.3	0.6	none	3.75	0.75
2019	8xBTHWA	2	0.25	0.6	none	3.9	0.78
2019	8xBTHWA	3	0.2	0.5	none	3.9	0.78
2019	8xBTHWA	4	0.3	0.4	none	3.85	0.77
2020	8xBTHWA	1	0.15	none	0.2	3.35	0.67
2020	8xBTHWA	2	0.25	none	0.15	3.5	0.7
2020	8xBTHWA	3	0.1	none	0.15	3.5	0.7
2020	8xBTHWA	4	0.2	none	0.3	3.6	0.72
2019	4xBTHWA	1	0.15	0.45	none	3.45	0.69
2019	4xBTHWA	2	0.2	0.4	none	3.5	0.7
2019	4xBTHWA	3	0.25	0.4	none	3.35	0.67
2019	4xBTHWA	4	0.25	0.4	none	3.4	0.68
2020	4xBTHWA	1	0.1	none	0.15	2.95	0.59
2020	4xBTHWA	2	0.1	none	0.2	3.2	0.64
2020	4xBTHWA	3	0.2	none	0.15	3.15	0.63
2020	4xBTHWA	4	0.15	none	0.15	2.95	0.59

Values were calculated as a mean value for each block consisting of 20 replicated each, for each tested variant of treatment
